# Letter regarding “Impact of screen use on behavior and sleep in patients with autism spectrum disorder”

**DOI:** 10.1055/s-0046-1820104

**Published:** 2026-06-23

**Authors:** Marcos Vinicius Sousa Varão, Guilherme Nobre Nogueira

**Affiliations:** 1 Universidade Federal do Ceará, Fortaleza CE, Brazil.; 2 Universidade Federal do Ceará, Hospital Universitário Walter Cantídio, Fortaleza CE, Brazil.

Dear Editor,

We read with great interest the review article by Lima et al. 1 (“Impact of screen use on behavior and sleep in patients with autism spectrum disorder”), which was recently published in *Arquivos de Neuro-Psiquiatria* . The authors address a highly-relevant and timely topic, given the exponential growth in screen exposure in childhood and the particular vulnerability of children with autism spectrum disorder (ASD) to environmental influences. However, there are some topics that need further discussion.

The review lies in its comprehensive synthesis of recent international evidence drawn from diverse geographic and sociocultural contexts, encompassing studies conducted across multiple continents. This global perspective is particularly important in the field of autism research, as patterns of screen use, access to digital technologies, parental practices, and healthcare infrastructures vary substantially worldwide and may influence behavioral and sleep-related outcomes.

The article appropriately distinguishes between passive screen exposure and potentially-beneficial uses. This balanced approach avoids oversimplification and aligns with real-world clinical observations that screens may serve both maladaptive and supportive roles in ASD management. Emerging evidence suggests that these qualitative factors may be as influential as exposure duration itself, particularly in children with ASD. Structured digital interventions and mediated serious games have been shown to exert positive effects on socioemotional skills, communication, and motor behaviors in children with ASD, indicating a potential therapeutic role for digital media when properly designed, supervised, and contextually integrated. 2 Beyond skill acquisition, such approaches may enhance engagement, motivation, and opportunities for social interaction when incorporated into clinical or educational settings. Incorporating these qualitative variables into future research would enable a more refined understanding of which forms of screen exposure may mitigate, rather than exacerbate, core ASD-related symptoms across socioemotional, communicative, and behavioral domains.

We noted that the bibliographic search was restricted to two databases (Medline/PubMed and SciELO) and used very specific descriptors ( *screen time* and *smartphone* ). In systematic or integrative reviews on medicine, the absence of databases such as Embase, Scopus, or Cochrane, as well as the non-inclusion of related terms (such as *social media* , *tablet* , *television* , *digital media* , and *gaming* ), may have restricted the number of studies retrieved, which is reflected in the initial number of only 72 articles identified. A more sensitive search strategy could capture a wider range of evidence that might use different terminologies to describe digital exposure.

Furthermore, the article lacks a formal risk of bias assessment. Although the review provides a valuable synthesis of recent evidence, the lack of a structured evaluation of methodological quality limits the strength of the conclusions. Without a systematic appraisal of selection bias, measurement bias, and confounding, it remains challenging to determine the extent to which the reported associations are valid.

Moreover, the review groups range from preschool children (approximately 3 years old) to adolescents (up to 21 years old). While this breadth is necessary for an overview, it tends to mask critical developmental nuances, as the impact of screens in early childhood operates through distinct mechanisms. As evidenced by Kerai et al. (2022), 3 excessive screen use (greater than 1 hour/day) at this specific stage compromises fundamental domains of school readiness, such as social competence and emotional maturity, by displacing vital activities such as free play and family interactions.

In summary, the review makes a valuable contribution by consolidating contemporary, globally-diverse evidence on the complex relationship involving screen use, behavior, and sleep in children with ASD. By highlighting the potential risks and existing knowledge gaps, the article provides an important foundation for future research and clinical guidance.
